# Candidate Genetic Markers of Schizophrenia Based on Exome Sequencing Data and Its Relation to Immunological, Clinical, and Morphometric Changes in the Russian Population

**DOI:** 10.17691/stm2025.17.2.06

**Published:** 2025-04-30

**Authors:** S.M. Rastorguev, S.V. Tsygankova, M.A. Kaidan, V.L. Ushakov, I.K. Malashenkova, S.A. Krynskiy, D.P. Ogurtsov, N.A. Hailov, N.V. Zakharova, G.P. Kostyuk

**Affiliations:** Senior Researcher, Department of Mental Disorders in Neurodegenerative Diseases of the Brain; Alekseev Psychiatric Clinical Hospital No.1, Moscow Department of Health, 2 Zagorodnoe Shosse, Moscow, 117152, Russia; Senior Researcher; Pirogov Russian National Research Medical University, 1 Ostrovitianov St., Moscow, 117513, Russia; Head of the Laboratory of Eukaryotic Genomics, Kurchatov Genome Center; National Research Center “Kurchatov Institute”, 1 Akademika Kurchatova Square, Moscow, 123182, Russia; Leading Specialist, Laboratory of Epigenetic Regulation of Hematopoiesis; National Medical Research Center for Hematology, Ministry of Health of the Russian Federation, 4 Novy Zykovsky Proezd, Moscow, 125167, Russia; Junior Researcher, Laboratory of Fundamental Research Methods, Scientific and Clinical Research Center of Neuropsychiatry; Alekseev Psychiatric Clinical Hospital No.1, Moscow Department of Health, 2 Zagorodnoe Shosse, Moscow, 117152, Russia; Associate Professor, Leading Researcher, Institute for Advanced Brain Studies; Institute for Advanced Brain Research, Lomonosov Moscow State University, 27/1 Lomonosov Prospect, Moscow, 119192, Russia; Senior Researcher, Department of Mental Disorders in Neurodegenerative Diseases of the Brain, Scientific and Clinical Research Center of Neuropsychiatry; Alekseev Psychiatric Clinical Hospital No.1, Moscow Department of Health, 2 Zagorodnoe Shosse, Moscow, 117152, Russia; Senior Researcher; National Research Nuclear University MEPhI, 31 Kashirskoe Shosse, Moscow, 115409, Russia; Head of the Laboratory of Molecular Immunology and Virology; National Research Center “Kurchatov Institute”, 1 Akademika Kurchatova Square, Moscow, 123182, Russia; Senior Researcher, Laboratory of Clinical Immunology; Federal Research and Clinical Center of Physical-Chemical Medicine, Federal Medical Biological Agency of Russia, 1A Malaya Pirogovskaya St., Moscow, 119435, Russia; Senior Researcher, Department of Epidemiology and Prevention of Mental Disorders, Scientific and Clinical Research Center of Neuropsychiatry; Alekseev Psychiatric Clinical Hospital No.1, Moscow Department of Health, 2 Zagorodnoe Shosse, Moscow, 117152, Russia; Senior Researcher, Laboratory of Cell Biology, Molecular Medicine and Immunology; National Research Center “Kurchatov Institute”, 1 Akademika Kurchatova Square, Moscow, 123182, Russia; Junior Researcher, Department of Mental Disorders in Neurodegenerative Diseases of the Brain, Scientific and Clinical Research Center of Neuropsychiatry; Alekseev Psychiatric Clinical Hospital No.1, Moscow Department of Health, 2 Zagorodnoe Shosse, Moscow, 117152, Russia; Senior Researcher, Laboratory of Cell Biology, Molecular Medicine and Immunology; National Research Center “Kurchatov Institute”, 1 Akademika Kurchatova Square, Moscow, 123182, Russia; Researcher, Laboratory of Clinical Immunology; Federal Research and Clinical Center of Physical-Chemical Medicine, Federal Medical Biological Agency of Russia, 1A Malaya Pirogovskaya St., Moscow, 119435, Russia; Senior Researcher, Resource Center for Molecular and Cellular Biology; National Research Center “Kurchatov Institute”, 1 Akademika Kurchatova Square, Moscow, 123182, Russia; Leading Expert, International Scientific and Educational Center of Neuropsychiatry; Samara State Medical University, 89 Chapayevskaya St., Samara, 443099, Russia; Leading Researcher; V.M. Bekhterev National Medical Research Center for Psychiatry and Neurology, Ministry of Health of the Russian Federation, 3 Bekhtereva St., Saint Petersburg, 192019, Russia; Professor, Chief Physician; Alekseev Psychiatric Clinical Hospital No.1, Moscow Department of Health, 2 Zagorodnoe Shosse, Moscow, 117152, Russia

**Keywords:** schizophrenia, exome sequencing, single nucleotide polymorphisms, olfactory receptors, magnetic resonance imaging, immunity, major histocompatibility complex, ankyrins

## Abstract

**The aim of the study** was to identify genetic markers of schizophrenia based on exome sequencing data, as well as to specify its potential relation to clinical manifestations of the disease, morphological changes in the brain and immune disorders.

**Materials and Methods:**

The analyzed sample consisted of 48 patients (23 men and 25 women; average age — 31.5±7.7 years) having a confirmed diagnosis of paranoid schizophrenia.

**Results:**

140 genes with differential polymorphisms and enriched categories that may be related to the pathogenesis of schizophrenia were identified. Analysis of genes with differential frequencies of functionally significant common single nucleotide polymorphisms (SNPs) by their major functions showed that the most common were genes involved in regulation of immune system functions and development of the nervous system, as well as genes being structural components of neurons and glia involved in the perception of sensory stimuli. The findings confirm the complexity of the genetic basis of schizophrenia. Analysis of the top 10 genes containing the most differential polymorphisms specifies such genes related to schizophrenia as *MUC12* and *SH3KBP1*. The genes involved in regulation of the immune response include *HLA-DQB2* which is one of the most significantly different SNPs between the group of patients and the general population; *HLA-DQB2* SNP (rs9276572) in patients is related to the signs of dysfunction of the antiviral component of immune system, structural changes in the brain and cognitive challenges. Although most of the detected genes are unique to the sample studied, additional studies are required to confirm these genes’ involvement in the pathogenesis of this disease as well as to identify the mechanisms of the disease onset and development. The rs9276572(C) polymorphism of *HLA-DQB2* requires further study as a new potential marker of immunological disorders, morphometric changes in the brain and cognitive impairment in schizophrenia. The data obtained indicate the need for personalized medicine, because the majority of genetic prerequisites are patient-specific and highlight the importance of further research to understand the genetic aspects of schizophrenia and develop innovative approaches to its diagnosis and treatment.

## Introduction

Schizophrenia is still one of the most mysterious and complex mental disorders that affects the lives of millions of persons worldwide. Despite a fairly high degree of heritability, which ranges from 50 to 80%, the mechanisms of its development are poorly understood due to its multifactorial and polygenic nature [1–4]. Genomic studies of the last decades identified about 300 common genetic variants (by means of genome-wide association studies) and more than 20 rare variants (by means of exome sequencing and copy number variant studies) related to the risk of schizophrenia development. In parallel, functional genomic and neurobiological studies provided detailed information about brain cells (numerous types of neurons, interneurons in various brain regions) and their changes in different variants of schizophrenia. Jointly, these results reflect the complexity of schizophrenia mechanisms, pointing to gene ensembles (polygenicity) in the disease pathogenesis and not to a single gene involvement [5]. A significant part of genetic contribution to schizophrenia development is given to ultra-rare and unique alleles that affect genes related to development of the nervous system and other important functions of the body [6]. Here, genes with common single nucleotide polymorphisms (SNPs) interact with genes containing *de novo* mutations in the protein-protein interaction network, which assumes mutual influence of both common and rare SNP variants in schizophrenia [7]. Considering modern technological advances in genetic studies, the use of various options for genomic sequencing is a key tool for a deeper understanding of the genetic basis of schizophrenia [8, 9].

Exome sequencing — being an analysis of the exome or a part of the genome that contains proteincoding DNA regions — provides opportunities for studying genetic factor basis of schizophrenia. As schizophrenia pathogenesis includes both genetic and environmental factors, the versatile nature of this disorder is taken into account. Exome sequencing allows for a comprehensive coverage of the genetic basis, identifying gene variants predisponent to schizophrenia development and revealing the relation of such gene variants to various forms of the disease [10]. Currently, it is generally accepted that schizophrenia pathogenesis is related to rare genetic polymorphisms that make a major contribution to schizophrenia and common polymorphisms with a small effect size [11].

Epidemiological and clinical data attend to the fact that some patients with schizophrenia have pronounced immune disorders and signs of systemic inflammation. Advances in neuroimmunology gave new insights into the mechanisms by which the immune system influences brain development and function, producing a variety of hypotheses about the potential role of the immune system in schizophrenia. Neuronal autoantibodies, microglia priming, and changes in the balance of T-cell subpopulations in the central nervous system (CNS) are thought to be immune mechanisms predisponent to the disease. Systemic inflammation and chronic latent infections may be relevant for the development and maintenance of immune disorders in the CNS [12, 13].

One should highlight a number of polymorphisms of immune response genes in genetic factors influencing the risk of schizophrenia. For instance, SNPs of genes encoding major proinflammatory and anti-inflammatory cytokines, including interleukins (IL) IL1-β, IL-6, IL-6R, IL-10, IL-17A, and tumor necrosis factor α (TNF-α), are related to schizophrenia [14–16]. Individual SNP alleles of genes involved in the regulation of neuroimmune interactions have been proved to be related to schizophrenia, including the brain-derived neurotrophic factor (BDNF) gene association with clinical manifestations of schizophrenia [17]. Studies of the genetic overlap between schizophrenia and immune- mediated disorders are also of definite interest in relation to the immunogenetic architecture of schizophrenia [18].

The era of genomics provides new opportunities for assessment of the immunological hypothesis of schizophrenia by using large-scale genetic data. Based on the results of genome-wide association studies [12], candidate immune response regulatory genes related to development of schizophrenia were identified. One should note that these include genes (*CD14*, *CLU*, *DPP4*, *NGF1-A*, *HSPD1*) that are expressed both in immune cells and in the brain, which suggests their dual role in immunity and CNS functioning [12]. A clear example is also the *C4* complement component gene, which encodes the protein responsible for immunological mechanisms of pathogenes removal and for synaptic pruning in the CNS. A number of this gene polymorphisms are associated with the risk of schizophrenia [19].

To specify the role of immunogenetic factors in development of clinically significant course of schizophrenia, one must determine both common and rare polymorphisms of the immune response genes (the frequency of which differs most significantly in patients with schizophrenia and healthy individuals) as well as study SNPs associations with immunological disorders, clinical characteristics, and structural changes in the patients’ brains.

**The aim of this study** was to search for genetic markers of schizophrenia based on exome sequencing data, as well as to identify their potential relation to clinical manifestations of the disease, morphological changes in the brain, and immune disorders.

This study is important in several ways: firstly, to determine genetic markers that can become a tool for preliminary screening of individuals with an increased predisposition to mental disorders; secondly, to identify molecular mechanisms that form the basis of the disease, which is necessary for a deeper understanding of schizophrenia pathogenesis and development of effective treatment strategies.

As the studied disease depends on many rare alleles, the study of a particular ethnicity is of a separate value. Such complex diseases as schizophrenia are characterized by patient-specific pathogenesis, while each case is important for understanding the general mechanisms of the disease. Thus, this publication is a step forward to personalized medicine, widening the possibilities of timely detection of the disease and therapeutic intervention for persons at risk of schizophrenia. The author hopes that the results of this study are not only beneficial for our understanding of the genetic aspects of schizophrenia, but also create new perspectives for development of innovative approaches to diagnosis and treatment of this severe mental disorder.

## Materials and Methods

###  

#### Selection and description of patients

The study was approved by the Independent Multidisciplinary Committee for Ethical Review of Clinical Trials (protocol No.12 dated July 14, 2017).

The studied sample included 48 patients (23 males and 25 females; average age — 31.5±7.7 years) who were repeatedly hospitalized in the all-day care departments of the Alekseev Psychiatric Clinical Hospital No.1 of the Moscow Department of Health (Russia) due to exacerbation of psychotic symptoms of the diagnosed paranoid schizophrenia (ICD-10 code: F20.0) during the period from 2019 to 2021. The study was conducted in accordance with the Declaration of Helsinki (2013). All patients provided their written informed consent after a full description of the study procedures.

Inclusion criteria: mental state at the time of examination must meet the criteria for schizophrenia according to ICD-10; provision of informed consent to participate in the study.

Exclusion criteria: severe somatic diseases in the decompensation stage; acute infectious diseases, immunoinflammatory and autoimmune diseases in the exacerbation phase; signs of psychoactive substance and alcohol abuse; pregnancy; refusal to participate.

Clinical and dynamic parameters (M±σ) of the study participants: duration of the disease from the onset of prodromal symptoms — 12.9±6.7 years; duration of the disease from manifestation — 6.6±5.1 years. Psychometric parameters upon admission (M±σ): PANSS (Positive and Negative Symptoms Scale for Schizophrenia; total score) — 87.26±24.32 points; PANSS P (severity of productive symptoms) — 20.76±7.40 points; PANSS N (severity of negative symptoms) — 25.24±6.84 points; PANSS G (severity of other mental disorders according to the general psychopathology scale) — 39.96±12.77 points; BFCRS (Bush-Francis Catatonia Rating Scale) — 4.61±5.06 points.

Study design: cross-sectional, observational.

#### DNA extraction

DNA was extracted from the peripheral blood of schizophrenia patients using QIAamp DNA Blood Mini Kits (QIAGEN, USA) in line with the manufacturer’s recommendations.

The concentration of the extracted DNA was measured on a Qubit 2.0 fluorimeter (Thermo Fisher Scientific, USA) using the Qubit dsDNA BR Assay Kit (Thermo Fisher Scientific, USA).

#### Preparation of Illumina libraries, sequencing

Libraries for exome sequencing were prepared using the KAPA HyperExome Kit (Roche, USA) according to the user manual.

150 ng of genomic DNA were fragmented on a Covaris S220 device (Thermo Fisher Scientific, USA), after which specific KAPA Universal Adapters were ligated to the ends of the obtained fragments using the KAPA HyberPrep Kit (Roche, USA).

Then, there was a two-step selection of fragments in the length range of 200–350 bp using AMPureXP Beads magnetic particles (Beckman Coulter, USA). The resulting fragments were amplified using primers specific to the adapters and hybridized with biotinylated probes to the target regions of the KAPA HyberCapture Reagent Kit (Roche, USA) for 28 h at 47°C. Biotinylated DNA probe hybrids were isolated and purified with streptavidin-conjugated magnetic particles, and a second amplification was performed. In order to remove non-target amplification fragments and adapter dimers, the DNA library was purified using AMPureXP Beads (Beckman Coulter, USA). The final concentration and validation of the prepared library were determined on the Qubit fluorimeter (Life Technologies, USA) using the Qubit dsDNA HS Assay Kit (Thermo Fisher Scientific, USA) and on the 2100 Bioanalyzer (Agilent Technologies, USA).

Library sequencing was performed on the NovaSeq 6000 platform (Illumina, USA) using the S1 flow cell and NovaSeq 5000/6000 S1 Reagent Kit (Illumina, USA; 2×150 cycles).

#### Bioinformatic analysis of sequencing results

Illumina nucleotide reads were mapped to the human reference genome. Bowtie 2 v.2.4.4 was used for mapping [20]. An index database for the GRCh38 genome assembly (https://www.ensembl.org/Homo_sapiens/Info/Index) was created using the Bowtie2- build tool. The genomic sequences were annotated into genes based on the annotation from the Ensembl database [21] (https://ftp.ensembl.org/pub/release-110/gff3/homo_sapiens/Homo_sapiens.GRCh38.110.chr.gff3.gz) using the BEDTools v.2.30.0 tool [22]. Mapping was performed using the very-sensitive-local parameter set. Compression, sorting, and indexing of the aligned sequences were performed using the SAMtools v. 1.16.1 package [23].

The bcftools v. 1.13–1 tool was used to identify SNPs from the aligned sequences [24].

The obtained variants were annotated using the SIFT 4G Annotator (Sorting Intolerant From Tolerant, SIFT) tool [25]. SIFT provided functional predictions for the detected polymorphisms in the form of TOLERATED (harmless) and DELETERIOUS (harmful) variants. A common problem for such studies is the large number of polymorphisms (and, thus, genes) that show a significant difference in allele frequency between the experimental group and the control group. In order to reduce the final number of genes, fairly strict filtering conditions for SNP loci were applied. For further work, only those loci that contained a homozygous genotype for the allele predicted by the SIFT algorithm as DELETERIOUS were selected. Then, for SNP loci that were included into the dbSNP database (https://www.ncbi.nlm.nih.gov/snp/), the population allele frequencies were determined according to the SNP allele frequency database for the European population (SAMN10492695) from NCBI (http://ftp.ncbi.nih.gov/snp/population_frequency/latest_release/freq.vcf.gz). The dataset (version build_ id=20201027095038) contained generalized data of 163,190 persons; these data were compared with the allele frequencies in the studied sample of 48 patients with schizophrenia. The statistical significance of the difference in allele frequencies was assessed using the poisson.test function from the standard set of functions of the R statistical programming environment (https://www.r-project.org/). The NOVEL-predicted loci (not in dbSNP) were searched for homozygous SIFT- predicted DELETERIOUS allele which are seen in the studied sample in at least 8 (out of 48) patients with schizophrenia.

Using the SIFT software, for the so selected loci the genes in which these loci were located were defined. Functional analysis and annotation of GO (Gene Ontology) categories, as well as category enrichment analysis, were performed using the DAVID web service [26].

#### Specification of brain morphometric parameters

The study of brain morphometric parameters and immunological parameters (lymphocyte subpopulation ratio, serum cytokine, chemokine, and growth factor levels) included 29 of 48 patients with schizophrenia (14 males and 12 females; average age — 31.52±2.17 years) who underwent exome sequencing and in-depth assessment of clinical symptoms; and 29 healthy volunteers comparable with the patients by age and gender. MRI scanning was performed on a MAGNETOM Verio 3T magnetic resonance tomograph (Siemens GmbH, Germany). A 32-channel brain coil was used to obtain the data. The morphometric study of the gray matter, high-resolution anatomical data were obtained for each patient based on a T1-weighted sequence: TR (time between two radiofrequency pulses) — 1900 ms; TE (interval between the radiofrequency pulse and the peak of the signal (echo) induced in the coil) — 2.21 ms; 176 slices; voxel size — 1×1×1 mm^3^. All obtained structural images were analyzed using the FreeSurfer software.

#### Determination of lymphocyte subpopulations

Determination of lymphocyte subpopulations was performed by flow cytofluorimetry using monoclonal antibodies. For cell staining, monoclonal antibodies for TBNK immunophenotyping (Becton Dickinson, USA) to the differentiation antigens CD3, CD16, CD56, CD45, CD4, CD19, CD25, CD8, CD127, labeled respectively with FITC (fluorescein isothiocyanate), PE (phycoerythrin), PerCP-Cy™5.5 (peridinin-chlorophyll protein complex with cyanine 5.5), PE-Cy™7 (PE complex with cyanine 7), APC (allophycocyanin), APC- Cy™7 (APC complex with cyanine 7), Alexa Fluor® 647 were used.

#### Multiplex analysis

For multiplex analysis, at the sample preparation stage, test tubes with gel and SiO_2_ coagulation activator were centrifuged for 20 min at 2000 rpm. After centrifugation, the obtained serum was aliquoted in 0.6 ml portions and frozen at 20°C below zero until analysis. Merck reagent kits (USA) were used to conduct multiplex analysis to determine serum proteins, including cytokines, chemokines, and growth factors.

#### Statistical data processing

Statistical processing of immunological, morphometric, and clinical study data was performed using standard Excel (Microsoft, 2010), Statistica 10.0 (Stat Soft, USA) application software packages. Standard statistical methods were used to process the results. Mean values (M), standard deviation (σ), and 95% confidence intervals were used to describe the data. The normality of distribution was assessed using the Shapiro–Wilk test. The Student’s t-test was used to assess differences in values between groups. Differences were considered statistically significant at p<0.05.

## Results

Over two and a half billion paired nucleotide reads of 150 nucleotides in length were received for all patients; the size of the sequenced fragments equaled to 500–700 nucleotides. The number of nucleotide reads for each DNA library, as well as the percentage of the mapped nucleotide reads to the reference genome are specified in Appendix 1.

After mapping and determination of SNP positions, 2,724,996 polymorphic positions for all 48 libraries were obtained (a position was considered polymorphic if at least one sample had an alternative SNP state). After annotation of nucleotide substitutions using the SIFT algorithm and selection of genotypes with a damaging substitution, 2337 SNP loci remained, and at least one patient from the studied sample of patients with schizophrenia had a homozygous genotype for such a damaging allele. As a result of differential analysis of allele frequencies between the studied sample and the general European population, 140 genes containing differential polymorphisms were selected.

The list of genes selected in line with the procedure described above is specified in Appendix 2. Despite a fairly strict filtering, a relatively large number of genes that differ in the studied sample were identified. However, this result was far below the number of genes usually identified in such studies.

In case of less strict filtering criteria, for example without the condition of damaging nature of the polymorphism or homozygous genotype, there would be a much greater number of such genes — even thousands of them, and it would be much more difficult to describe them.

One of the widely used methods to describe gene lists is the so-called GO analysis. It allows identifying gene categories that are enriched in the studied gene list, that is gene categories that are presented in a specific list significantly more often than in a random list of the same size. Such an analysis was performed for the genes isolated in the study group of patients. The results are provided in Table 1.

**T a b l e 1:** List of gene categories enriched in genes with disorders identified in the studied sample of patients with schizophrenia

Gene category	Number of genes	OR	p	FDR
Sensory transduction	22	11.9	1.4·10^-7^	8.8·10^-6^
Olfaction	17	9.2	2.3·10^-6^	6.9·10^-5^
Olfactory receptor activity	17	9.2	1.4·10^-6^	0.00035
Detection of chemical stimulus involved in sensory perception of smell	17	9.2	5.3·10^-7^	0.00036
Glycoprotein	65	35.1	3.7·10^-5^	0.00074
Olfactory receptor	17	9.2	2.2·10^-6^	0.00077
Olfactory transduction	17	9.2	5·10^-6^	0.00086
G-protein coupled receptor activity	21	11.4	1.1·10^-5^	0.0013
Transducer	22	11.9	6.1·10^-5^	0.0017
G-protein coupled receptor	21	11.4	6.1·10^-5^	0.0017
Receptor	33	17.8	0.00015	0.0027
TOPO_DOM: Extracellular	50	27	4·10^-6^	0.003
TRANSMEM: Helical	75	40.5	1.1·10^-5^	0.003
DOMAIN: G-protein coupled receptors family 1 profile	19	10.3	1.1·10^-5^	0.003
Integral component of membrane	71	38.4	3.7·10^–5^	0.0052
Plasma membrane	70	37.8	5.9·10^–5^	0.0052
Lumenal side of endoplasmic reticulum membrane	5	2.7	9.4·10^–5^	0.0052
Integral component of lumenal side of endoplasmic reticulum membrane	5	2.7	9.4·10^–5^	0.0052
Disulfide bond	51	27.6	0.0012	0.012
REPEAT: ANK	10	5.4	6.6·10^–5^	0.013
CARBOHYD: N-linked (GlcNAc...) asparagine	61	33	0.0001	0.016
COMPBIAS: Polar residues	85	45.9	0.00013	0.017
G protein-coupled receptor, rhodopsin-like, 7TM	19	10.3	0.00012	0.021
TRANSMEM: Helical; Name=7	21	11.4	0.00023	0.027
G protein-coupled receptor, rhodopsin-like	18	9.7	0.00027	0.032
MHC class II, beta chain, N-terminal	4	2.2	0.00048	0.037
70.Signal_peptides_(MHC class I molecules)	2	1.1	0.021	0.041
Odorant binding	7	3.8	0.0007	0.043
Peptide antigen binding	5	2.7	0.00071	0.043
TRANSMEM: Helical; Name=6	21	11.4	0.00059	0.047
TRANSMEM: Helical; Name=5	21	11.4	0.00064	0.047

N o t e. The list is sorted by the FDR (false discovery rate) value, which reflects the degree of enrichment of each gene category. The table presents only categories that are enriched at a significance level of 95% (FDR<5%). MHC — major histocompatibility complex; OR - odds ratio.

Analysis of genes with differential frequencies of functionally significant SNPs according to annotations (https://www.genecards.org/) showed that most of these genes can be combined into several categories according to their main functions:

immune system — 31;
development of the nervous system, structural components of neurons and glia — 29;
sensory receptors and regulators of sensory receptor activity — 19, including 14 olfactory receptors;
transcription factors — 12;
energy metabolism, mitochondrial proteins — 11;
proteins of microtubules, flagella and cilia — 7;
tumor antigens — 6;
epithelial barriers — 6;
DNA reparation and protein processing — 3;
unknown functions — 4.

The largest number of genes with differential expression were related to regulators of immune system functions: *AGAP1*, *APOH*, *ARHGEF40*, *CANX*, *CLEC2D*, *CLEC4M*, *COPS7A*, *DDRGK1*, *GBP3*, *HLA-B*, *HLA-DQB2*, *HSPA1L*, *ICOSLG*, *IRS4*, *KMT2E*, *MC1R*, *MMP27*, *NPLOC4*, *RNF213*, *RNFT2*, *RSRP1*, *SEMA4D*, *SH3KBP1*, *SPINK5*, *TMEM176B*, *TNXB*, *TPSB2*, *TRIM64C*, *TSPAN6*, *UBASH3A*.

The analysis of gene categories also demonstrated that a significant number thereof, including highly enriched ones, was related to olfactory receptors. A connection between olfactory receptors and the development of schizophrenia is possible: for example, due to changes in the perception of the environment and social cues. However, this gene category is often distinguished during the search for differential genes and outliers in various genomic studies. Apparently, this is a rather dynamic genetic category, subject to rapid and easy changes. Moreover, due to the use of strict criteria for filtering and selecting damaged genes, one can state that olfactory receptor genes are more suitable for such conditions, as a defect in such a gene does not significantly affect the sapience health. At that, numerous studies provided evidence of abnormal olfactory function in schizophrenia and other psychotic disorders [27]. Of all the senses, olfaction is most closely related to the frontal and temporal lobes of the brain; these are regions involved in affective and mnemonic functions and schizophrenia pathogenesis. In patients with schizophrenia, impairment of odor identification and discrimination, changes in the olfactory sensitivity threshold, and olfactory memory impairment were seen, but the causes of olfactory disorders in schizophrenia are still unclear [28].

In males at risk of psychosis development, bilateral reduction in the volume of the olfactory bulb, abnormal asymmetry of the posterior nasal cavities and olfactory grooves (the left groove decreased compared to the right one), and a decrease in the volume of gray matter in the olfactory cortex on the left (not on the right) were seen. In females at risk of psychosis development, similar changes were noted, but less frequently and to a lesser extent. The volume of the left olfactory bulb in both groups correlated with negative symptoms [29].

It should be noted that the olfactory epithelium has an important function of an immune barrier and is involved in neuroimmune interactions [30]. There are almost no studies devoted to the genetic aspects of olfactory dysfunction in schizophrenia, so further studies in this area are of great interest.

The Figure shows the results of functional enrichment for the above mentioned list of genes. The analysis proved that the enrichment in the category of “peptide antigen binding” had the greatest statistical significance in the list. Binding of antibodies to peptide antigens of pathogens is required for their neutralization and removal. Moreover, enrichment in such categories as “binding to ligase of biquitin-like proteins”, “luminal side of the endoplasmic reticulum membrane” and “outer side of the cytoplasmic membrane” was to be paid attention to and indicated a probable change in the activity of binding and presentation of peptide antigens, as well as proteasomal cleavage of intracellular proteins. This may result in a change in the processes of antigen-dependent and antigen-independent cytotoxicity, carried out by CD3^+^CD8^+^ cytotoxic T cells and CD3^–^CD16^+^CD56^+^ NK cells, respectively. Considering that a number of genes in the list have an inhibitory effect on the processes of antigen presentation and cytotoxicity (*CLEC2D*, *ICOSLG*, *MC1R*, *RNFT2*, *SPINK5*, *TMEM176B*, *UBASH3A*), one can assume that in a number of patients with schizophrenia, their functionally significant SNPs contribute to the chronicity of infections, systemic inflammation, as well as autoimmune reactions and damage to their own cells.

**Figure:**
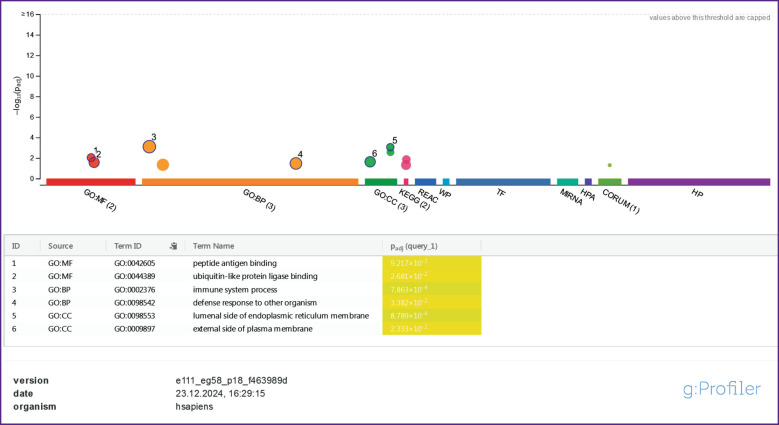
Results of functional enrichment of genes regulating immune processes and having differential frequencies of functionally significant SNPs in patients with schizophrenia

The most statistically significant differences in the frequency of functionally significant SNPs with the control group among the genes involved in the regulation of immune system functions were noted for the following genes: *CLEC2D* (p=0.00056), *IRS4* (p=0.00072), *HLA-DQB2* (p=0.0013). For instance, in 46.7 and 48% of patients, respectively, SNPs of the *CLEC2D* and *HLA-DQB2* genes were found in a homozygous state. The first belongs to the family of C-lectin receptors of NK cells, encodes the LLT1 protein, which is a ligand of the inhibitory receptor of NKR-P1A NK cells. HLA-DQB2 is a class II major histocompatibility complex antigen which is not expressed in monocytes and dendritic cells but is observed in Langerhans cells, where it can participate in the presentation of antigens to CD3^+^CD4^+^ T helpers [31]. The major histocompatibility complex genes have very dynamic nature and encode membrane proteins whose main functions are related to the presentation of antigen peptides to T cells, regulation of lymphocyte cytotoxicity, and inflammatory response. SNPs of the major histocompatibility complex genes affect the risk of a number of autoimmune and oncological diseases, and also determine the development of the graft vs host reaction during organ transplantation. Currently, the role of major histocompatibility complex proteins in development of the nervous system and neuroplasticity was also shown, mainly due to the regulation of the phenotype of microglial cells and, in particular, their function of synaptic pruning (removal of excess synapses to optimize neural networks), which can be impaired in schizophrenia [32].

Although the relation of allelic variants of the *HLA- DQB2* gene to the development of graft vs host reaction was not proved, the level of expression of HLA-DQB2 mRNA and protein and the development of kidney transplant rejection are somehow linked [33, 34]. High levels of this gene expression in breast cancer are a potential marker of a favorable prognosis [35].

Table 2 provides the following results: assessment of the parameters of cellular immunity; level of the CXCL10 chemokine, involved in the activation of the Th1 component of immunity; IL-1RA and TGF-α immunoregulatory proteins; vascular endothelial growth factor (VEGF) in patients with schizophrenia depending on the rs9276572(C) SNP of the *HLA- DQB2* gene and in the group of healthy volunteers. The G allele, which was found in 15 of 29 patients who underwent immunological analysis and studies of brain morphometric parameters (in total, the G allele was detected in 20 of 48 patients with schizophrenia included in the study), was related to changes in cellular immunity in schizophrenia, including an increase in the content of CD3^+^ T cells, CD3^+^CD8^+^ cytotoxic T cells, a decrease in the number of CD3^–^CD16^+^CD56^+^ NK cells (natural killers). CD3^+^CD8^+^ T cells and CD3^–^ CD16^+^CD56^+^ NK cells are effector cytotoxic cells that are major actors in removal of virus-infected and altered cells from the body. The functions of CD3^+^CD8^+^ T cells are antigen-dependent, whereas of CD3^–^CD16^+^CD56^+^ NK cells are antigen-independent.

**T a b l e 2:** Interrelations of the rs9276572 genetic polymorphism of *HLA-DQB2* with immunological and clinical parameters in patients, M±σ

Indicator	AA genotype	AG/GG genotype	Control
CD3^+^ T cells (%)	72.03±2.50 p=0.005*	78.94±3.26 p=0.006**	70.89±3.35
CD3^+^CD4^+^ T helpers (%)	44.83±3.71	47.34±4.02	42.43±2.74
CD3^+^CD8^+^ cytotoxic T cells (%)	23.68±3.38	28.06±2.83 p=0.034**	23.77±1.90
CD3^–^CD16^+^CD56^+^ NK cells (%)	13.16±2.65 p=0.032*	8.40±1.86 p=0.0008**	17.78±3.52
CD3^–^CD19^+^ B cells (%)	13.10±3.26	10.81±1.69	10.74±1.24
CXCL10 (pg/ml)	73.57±22.02 p=0.026*	42.70±12.32 p=0.038**	64.19±13.85
IL-1RA (pg/ml)	57.98±33.65 p=0.028*	15.08±6.48	27.14±16.22
TGF-α (pg/ml)	13.0±4.87	7.82±2.27	7.42±2.37
VEGF (pg/ml)	84.66±30.50 p=0.012*	33.17±17.17 p=0.009**	83.34±28.07
PANSS P2; conceptual disorganization (points)	2.77±0.38 p=0.006*	3.60±0.37	—
PANSS N5; difficulties in abstract thinking (points)	3.15±0.36 p=0.037*	3.73±0.36	—

* Statistically significant differences between the AA homozygote and other genotypes according to Student’s t-test (p<0.05); ** statistically significant differences with the control group according to Student’s t-test (p<0.05); PANSS P — severity of productive symptoms, PANSS N — severity of negative symptoms.

Moreover, patients with the G allele had decreased levels of the CXCL10 chemokine, which stimulates cytotoxicity reactions. Thus, such patients showed signs of impaired activity of antigen-dependent and antigen-independent cytotoxicity mechanisms. They also had decreased levels of VEGF, which may indicate endothelial dysfunction. VEGF is an important neuroprotective factor that reduces the damaging effects of neuroinflammation. *In vitro* studies demonstrated that VEGF protects neurons from oxidative stress, hypoxia, hypoglycemia, and glutamate-mediated excitotoxicity [36]. Furthermore, VEGF supports synaptic plasticity and promotes the growth, survival, differentiation, and migration of neuronal and glial cells, and is involved in the regulation of blood flow and angiogenesis in the central nervous system. The data confirm an inverse correlation between VEGF levels and frontal lobe volume in schizophrenia, thus one can assume that this protein has a protective effect on morphometric changes in the brain of patients [37].

According to the assessment of brain morphometric parameters, the G allele of the rs9276572(C) SNP of the *HLA-DQB2* gene is related to an expressed decrease in the cortex thickness in the temporal and frontal lobes of the brain, as well as in the calcarine sulcus of the occipital lobe (Table 3). At that, patients without this allele did not have a significant (p<0.01) decrease in the thickness of the cortex compared to the control group.

**T a b l e 3:** Interrelations of the rs9276572 genetic polymorphism of HLA-DQB2 with morphometric changes in the brain in patients with schizophrenia, M±σ

Indicator	AA genotype	AG/GG genotype	Control
Thickness of calcarine sulcus on the right (mm)	1.81±0.04 p=0.0015*	1.70±0.04 p=0.0009**	1.82±0.04
Thickness of insular cortex on the left (mm)	2.97±0.06 p=0.0039*	2.83±0.07 p=0.0004**	2.99±0.05
Thickness of inferior temporal gyrus on the right (mm)	2.74±0.06 p=0.0050*	2.61±0.06 p=0.0002**	2.77±0.04
Thickness of superior frontal gyrus on the left (mm)	2.62±0.05 p=0.0060* p=0.019**	2.52±0.04 p=0.0002**	2.70±0.03
Thickness of superior frontal gyrus on the right (mm)	2.68±0.06 p=0.0064*	2.56±0.05 p=0.00002**	2.73±0.03
Thickness of paracentral lobule on the left (mm)	2.41±0.07 p=0.0064*	2.29±0.05 p=0.00002**	2.45±0.04
Thickness of opercular part of the right inferior frontal gyrus (mm)	2.56±0.08 p=0.024**	2.46±0.05 p=0.000002**	2.67±0.05
Thickness of opercular part of the left inferior frontal gyrus (mm)	2.51±0.06 p=0.0074**	2.45±0.05 p=0.00002**	2.62±0.04

* Statistically significant differences between the AA homozygote and other genotypes according to Student’s t-test (p<0.05); ** statistically significant differences with the control group according to Student’s t-test (p<0.05).

Some patients with schizophrenia (6%) also had functionally significant polymorphisms of the *IRS4* gene, which: is involved in signal transmission of receptors with tyrosine kinase activity, including insulin receptors; stimulates the mitogenic effects of the IGFR1 receptor; regulates plastic metabolism and glucose metabolism. One should note that insulin-like growth factor (IGF-1), the main agonist of IGFR1, is involved in the regulation of neurogenesis, synaptogenesis, axon myelination, and dendritic branching. The author has previously demonstrated [38] that the IGF-1 level is related to the severity of immune-inflammatory disorders, morphometric disorders of the brain, and motor disorders (catatonia) in schizophrenia. There are data proving that the IGF-1 level is related to positive sensitivity to treatment in schizophrenia [39]. One should also note that the list of genes with differences in the frequency of functionally significant SNPs included a total of 11 genes regulating energy metabolism. Considering the obtained data, one can assume the role of genetically determined disorders of mitochondrial function, energy metabolism, and insulin signaling as well as immune changes related to impaired immunoregulatory functions of IGF-1 in the pathogenesis of some instances of schizophrenia.

Another way to analyze the NGS data is to select some genes that are the most differential for a specific sample of patients. The top 10 genes with the highest allele frequency differences between patients and the general European population were chosen. The list of these genes is provided in Table 4.

**T a b l e 4:** Genes that contain the most differential alleles in the sample of patients with schizophrenia

Gene	dbSNP ID	Chromosome	Position	Allele	Allele frequency in the European population	Number of homozygous genotypes in the patients’ sample
*ERICH6B*	rs117004691	13	45596602	С	0.0001539	15
*LRRC37A*	rs748643542	17	46330700	A	0.0752166	31
*TAS2R43*	rs68157013	12	11092126	G	0.2747180	21
*MUC12*	rs74373577	7	101004284	T	0.7006194	2
	rs111391555	7	101004313	C	0.5265895	4
	rs202107976	7	100995872	T	0.4892652	3
*POTED*	rs6517869	21	13610565	A	0.0	21
	rs200814491	21	13610400	G	0.0	1
*NCF4*	—	22	36875840	C	—	20
*ANKRD36*	rs150243949	2	97152527	T	0.0	5
*ACOT4*	rs35724886	14	73593804	A	0.0389513	4
	rs77814755	14	73593813	G	0.0	4
*SH3KBP1*	rs752483118	X	19588685	A	0.0	2
*RPGR*	rs12688514	X	38285569	T	0.0817302	6

N o t e. For some genes, multiple differential alleles were found, which is reflected in the dbSNP ID column. For the *NCF4* gene, the found differential locus was unique to the studied sample and was not included in the dbSNP database.

The SNP alleles in Table 4 are classified as damaging for the corresponding gene according to the SIFT algorithm. The majority of genes show an increased frequency of the mentioned damaging allele among persons with schizophrenia compared to the general European population. However, in case of the *MUC12* gene, the opposite trend is observed: the frequencies of the “damaging” allele in the population are quite significant and amount to 0.7 (rs74373577), 0.52 (rs111391555) and 0.48 (rs202107976) for different positions in the gene. At that, in the analyzed sample, only 2, 4, and 3 individuals (out of 48 patients), respectively, have a homozygous genotype for this allele. It should be noted that one shall square the allele frequency to calculate the theoretical frequency of the homozygous genotype.

There are several possible explanations for this phenomenon, such as a decrease in susceptibility to schizophrenia as a result of a breakdown of the corresponding gene or a possible incorrectgness of the SIFT algorithm with respect to this allele, which, most likely, does not violate the *MUC12* gene functioning. However, it is assumed that the most probable explanation is the availability of a defective copy of the *MUC12* gene in the reference sequence, accidentally included in the GRCh38 genomic assembly. And as the alternative alleles of the *MUC12* gene, shown in Table 4 in the Allele column, lead to significant changes in the protein structure (apparently, to a more correct structure), the algorithm interprets this phenomenon as a modification/damage. This hypothesis is indirectly supported by the fact that alternative amino acids (different from human beings) were found at this position in the MUC12 protein in other species (for exampole, in mice), while the surrounding amino acids are mainly identical.

## Discussion

Currently, there are several online resources that contain candidate genes involved in the pathogenesis of schizophrenia [40–42]. For example, the SZDB database (http://szdb.org/) [42] contains, inter alia, genes that were found during the analysis of exome sequencing of schizophrenia patient samples. There are 9366 genes contained in this database, which is more than a third of all protein-coding genes in humans. This number is based only on 16 studies of exome sequencing of patients with schizophrenia, although such works are being published more frequently.

The amount of identified genes suggests that each sample of patients contains a large number of unique polymorphisms that contribute to the development of schizophrenia in this group of patients. It is possible that almost any gene can contain an allelic variant which somehow affects the predisposition to schizophrenia or other complex diseases. This may provide that any study related to the search for genetic variants affecting susceptibility to schizophrenia is important to understand the mechanisms for the disease onset. As a result of accumulation of such information, people should understand which genetic changes are most important for the schizophrenia pathogenesis.

Every new study results in accumulation of knowledge and significantly contributes to widening the available information for identification of the molecular causes of the disease. In this regard, this study is also useful for studying schizophrenia and identifying genes related to the disease. Of the 140 genes identified in this study, only 102 are included into the SZDB database, the remaining genes are unique to the studied sample of patients. Moreover, out of the top 10 genes, only two — *TAS2R43* and *NCF4* — are available in the SZDB. This confirms that genetic causes of the disease are individual for each patient, thus something common in the mechanisms of predisposition is to be looked for at the upper levels of the genetic material.

The author applied strict filtering criteria to the obtained data. On the one hand, this allowed to significantly reduce the list of genes for processing, thus making further evaluation of the results easier, but on the other hand, it limited the possibilities for such types of analysis as GO category enrichment and functional annotation analysis, for which a large number of genes were suitable. The functional annotation analysis conducted in addition to the categories related to olfactory receptors (which can apparently be related to the disease) revealed only a few additional categories enriched in the gene list of patients. These categories include luminal side of the endoplasmic reticulum membrane, integral component of the luminal side of the endoplasmic reticulum membrane, major histocompatibility complex class I, and peptide antigen binding. These categories contain genes related to antigen presentation and immune response. The study list includes six genes of this kind: *HLA-DRB5*, *CANX*, *HLA-B*, *HLA-C*, *HLA-DQB2*, *CLEC4M*. A large number of studies provided examples of these genes involvement in pathogenesis of schizophrenia and other mental diseases. For example, recent studies [43–46] have shown that these genes are related to a predisposition to schizophrenia and to a response to antipsychotic drugs.

Annotation of functions of genes whose polymorphisms were found with differential frequency in schizophrenia allowed identifying a significantly larger number of genes related to immune system functions — over 30 genes. Functional enrichment of these genes indicates a change in the activity of processes related to antigen presentation, antigendependent, and antigen-independent cytotoxicity system. Antigen presentation impaired by perivascular macrophages and CNS microglial cells may be one of the pathogenetic mechanisms of schizophrenia, contributing to development and maintenance of chronic neuroinflammation and neuronal damage. This impairment may be due to the combined action of several factors, including latent viral infections and altered microglial reactivity due to excessive immune activation at early stages of development, as well as genetic predisposition. The latter is implemented, inter alia, due to variability of the genes of the major histocompatibility complex, which provide antigen presentation and affect the regulation of NK-cell functions and CD3^+^CD8^+^ cytotoxic lymphocytes. Currently, there are convincing evidence that allelic variants of *HLA* genes, including HLA-DQB1*0602 and HLA-DRB1*04, can affect the risk of schizophrenia [12]. However, the relation of the *HLA- DQB2* gene alleles with schizophrenia has not been previously studied. In this study, it is first shown that the rs9276572(C) SNP of this gene is seen in schizophrenia with increased frequency and is related to disorders of the cytotoxicity system. At that, the number of NK cells that provide for antigen-independent cytotoxicity, as well as the level of the CXCL10 chemokine, stimulating the chemotaxis of cytotoxic lymphocytes and NK cells, were significantly reduced. The analyzed group of patients was also characterized by a decrease in the VEGF level, regulating angiogenesis in the central nervous system and exerting neuroprotective functions. According to the structural MRI, these patients had an expressed decrease in the cortex thickness in the frontal and temporal lobes of the brain compared to patients without this allele and the control group. From a clinical point of view, the G allele carriers in the rs9276572 SNP locus of the *HLA-DQB2* gene were characterized by a greater severity of cognitive challenges according to the PANSS scale (PANSS P2 — conceptual disorganization, PANSS N5 — difficulties in abstract thinking).

Moreover, the study gene list contains several enriched categories related to ankyrins. Ankyrins are widely expressed adaptors that coordinate membrane proteins into specialized domains and link them to the submembrane cytoskeleton. Ankyrins were proved to be involved in organization of the axon initial segment and nodes of Ranvier in neurons. Ankyrins were identified in synapses, where they not only organized and stabilized neurotransmitter receptors but also modulated dendritic spine morphology and regulated adhesion to the presynaptic site. Ankyrin genes are closely related to nervous system pathologies and psychiatric disorders, including bipolar disorder, schizophrenia, and autism [47].

Analysis of the top 10 genes that contained the most differential SNP loci in the patient sample showed that in addition to the two genes presented in the SZDB database, two other genes were described as related to schizophrenia. In the study [48], the *MUC12* gene was included in the top 10 mutated genes in the research of early schizophrenia (childhood-onset schizophrenia). In the rat model study [49], it was shown that the *SH3KBP1* gene was important for the mechanisms of schizophrenia development, which were related to prenatal nutritional deficiency in the mother during pregnancy.

## Conclusion

This study which was conducted using exome sequencing methods, highlighted the importance of this method for identification of new genes related to schizophrenia. Most of the identified genes were unique to the studied sample and require further study and explanation. The rs9276572(C) *HLA-DQB2* polymorphism requires further study as a new potential marker of immunological disorders, morphometric changes in the brain, and cognitive impairment in schizophrenia.

In general, the study makes an important contribution to understanding the genetic basis of schizophrenia and highlights the complexity and diversity of factors influencing this disease. Further research in this area can help developing more effective diagnostic and therapeutic methods, as well as better understand the biological basis of mental diseases.
